# Supplementary material for: Metabolomics of Type 2 Diabetes Mellitus in Sprague Dawley Rats—In Search of Potential Metabolic Biomarkers
Source: Int J Mol Sci. 2023 Aug 5;24(15):12467. doi: 10.3390/ijms241512467 (PMC10419637; doi:10.3390/ijms241512467)
Supplement: Supplementary file 1 [file ijms-24-12467-s001.zip › ijms-2534544-Supplementary Figures and Tabels.pdf]

Supplementary figures and tables.

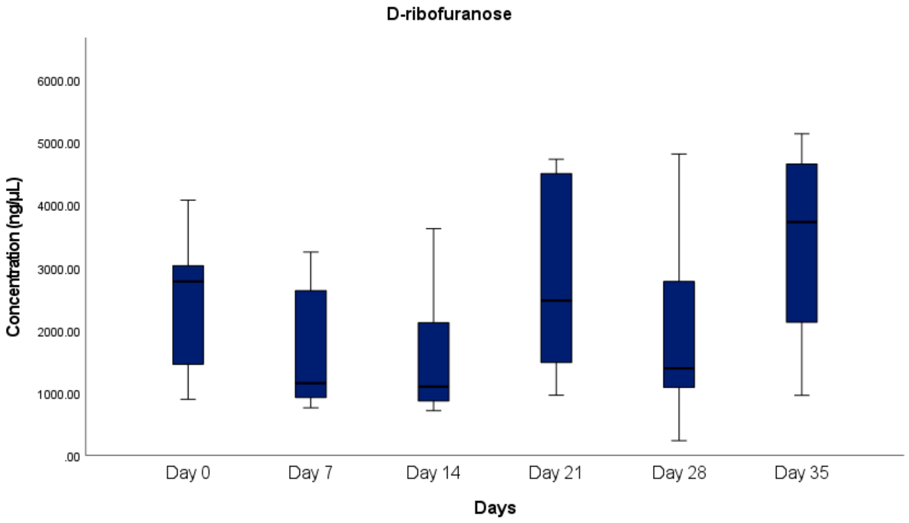

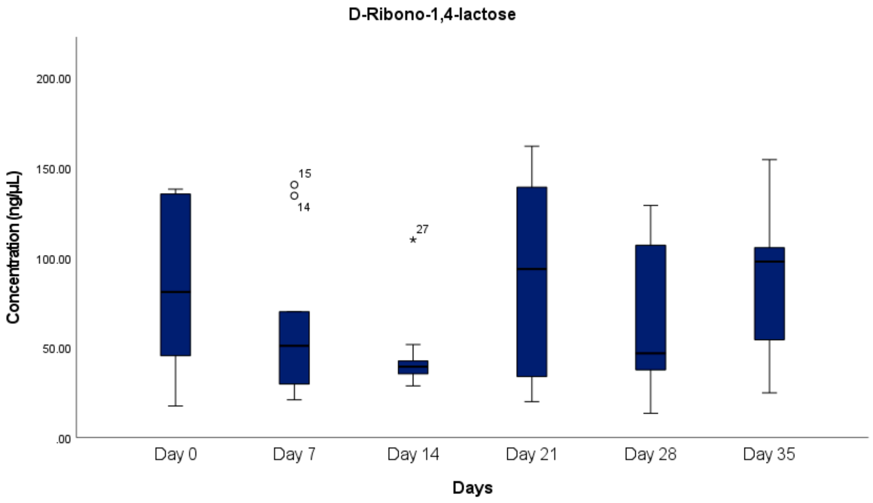

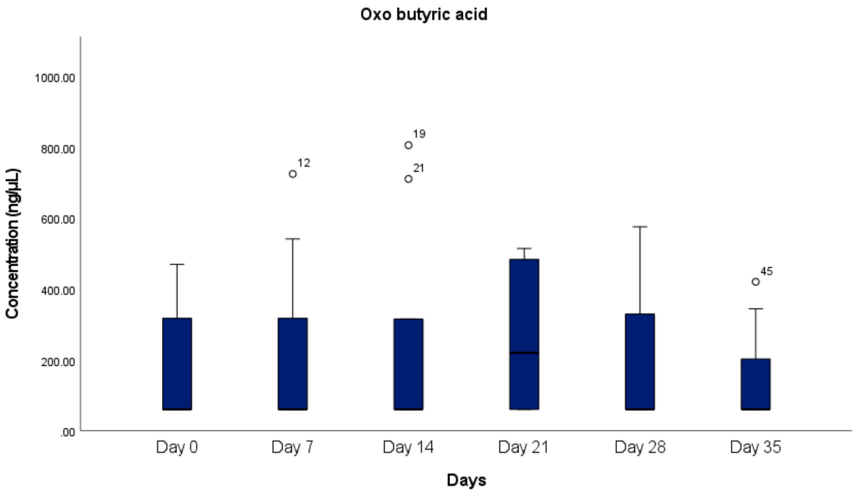

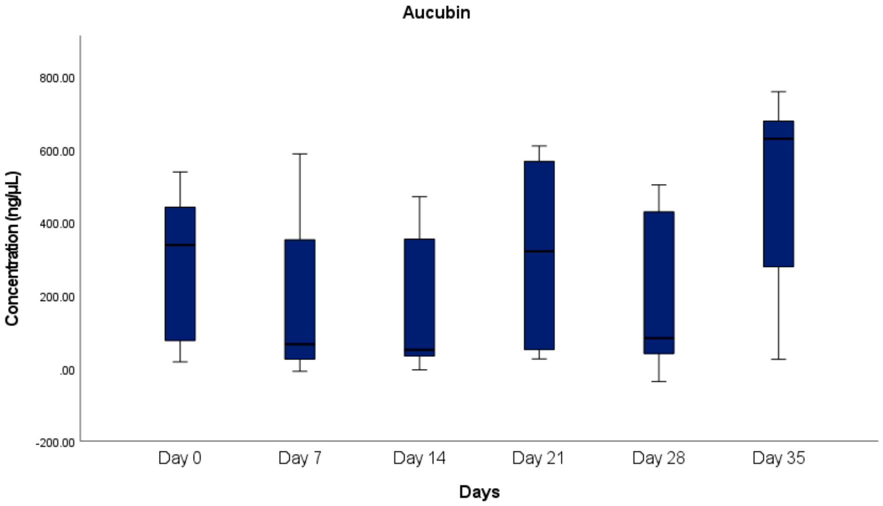
Aucubin
800.00
600.00
400.00
200.00
.00
-200.00
Concentration (ng/µL)
Day 0
Day 7
Day 14
Day 21
Day 28
Day 35
Days

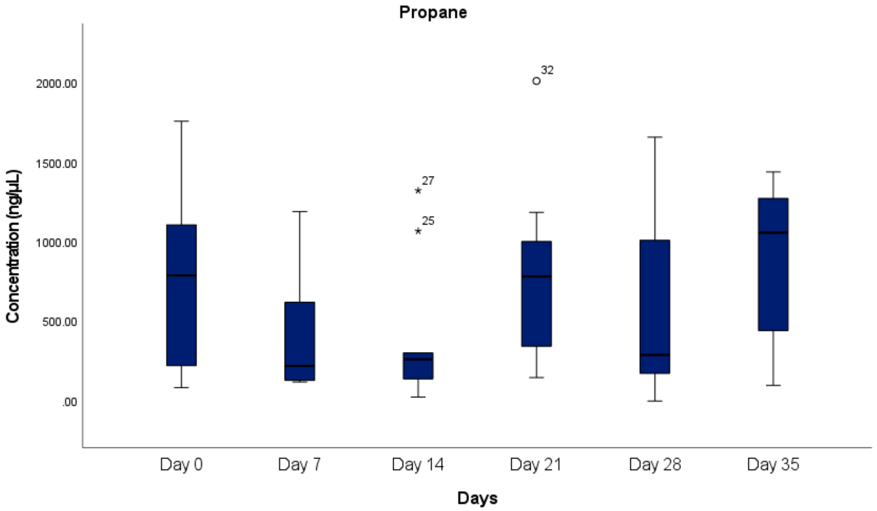
Propane
2000.00
1500.00
1000.00
500.00
.00
Concentration (ng/µL)
32
27
25
Day 0
Day 7
Day 14
Day 21
Day 28
Day 35
Days

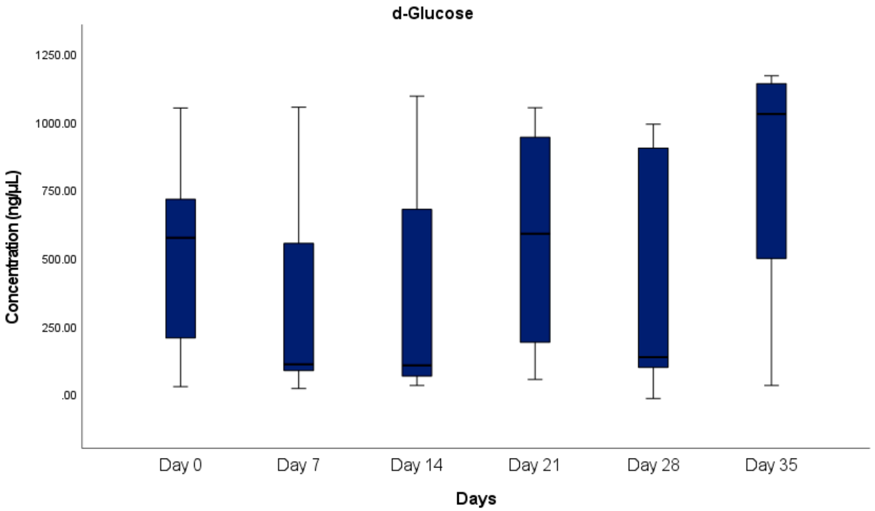
d-Glucose
1250.00
1000.00
750.00
500.00
250.00
.00
Concentration (ng/µL)
Day 0
Day 7
Day 14
Day 21
Day 28
Day 35
Days

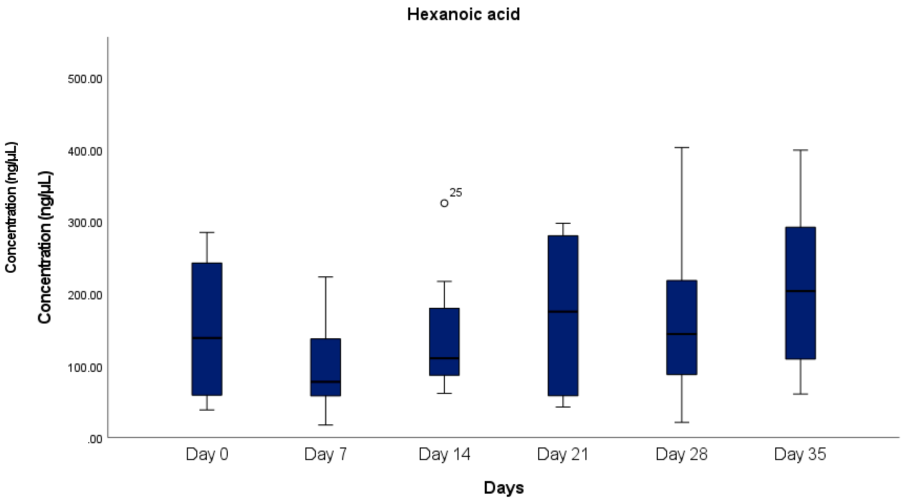
Hexanoic acid
500.00
400.00
300.00
200.00
100.00
.00
Concentration (ng/µL)
Concentration (ng/µL)
25
Day 0
Day 7
Day 14
Day 21
Day 28
Day 35
Days

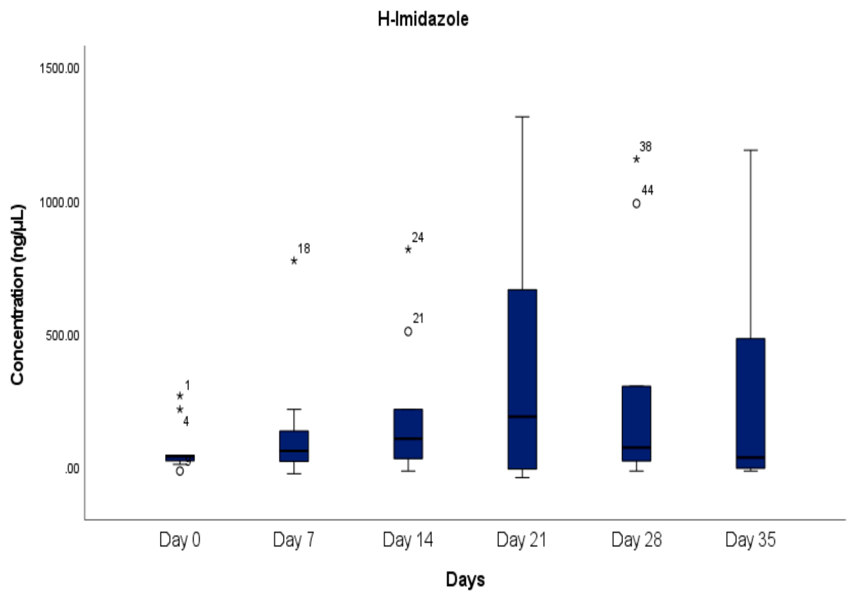
H-Imidazole
Concentration (ng/µL)
1500.00
1000.00
500.00
.00
1
4
18
24
21
38
44
Day 0
Day 7
Day 14
Day 21
Day 28
Day 35
Days

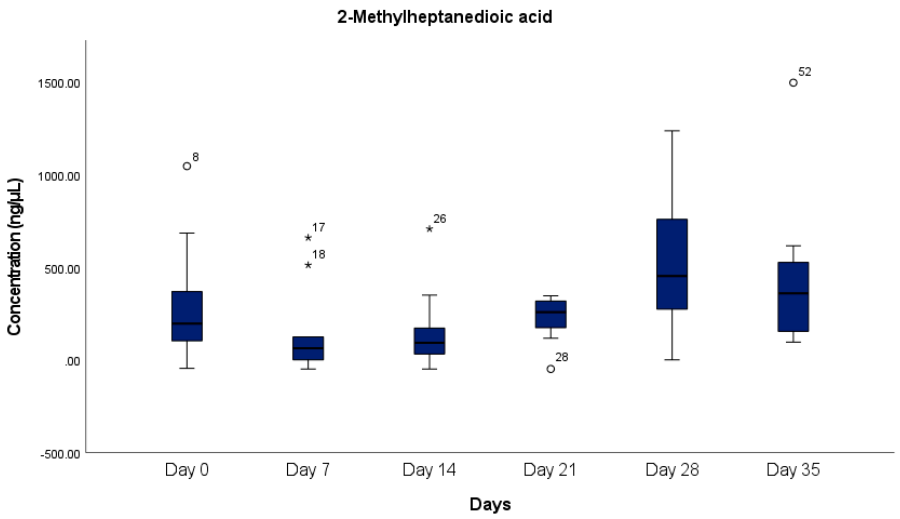
2-Methylheptanedioic acid
Concentration (ng/µL)
1500.00
1000.00
500.00
.00
-500.00
8
17
18
26
28
52
Day 0
Day 7
Day 14
Day 21
Day 28
Day 35
Days

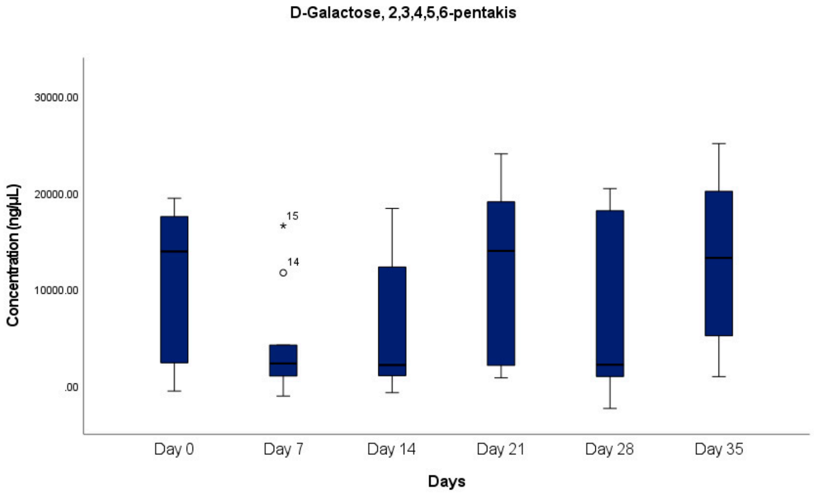
D-Galactose, 2,3,4,5,6-pentakis
Concentration (ng/µL)
30000.00
20000.00
10000.00
.00
15
14
Day 0
Day 7
Day 14
Day 21
Day 28
Day 35
Days

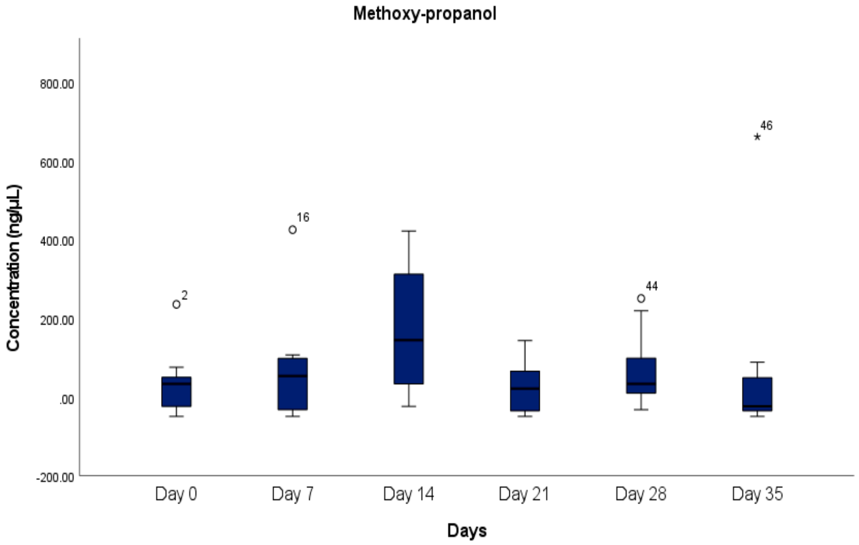
Methoxy-propanol
Concentration (ng/µL)
800.00
600.00
400.00
200.00
.00
-200.00
2
16
44
46
Day 0
Day 7
Day 14
Day 21
Day 28
Day 35
Days

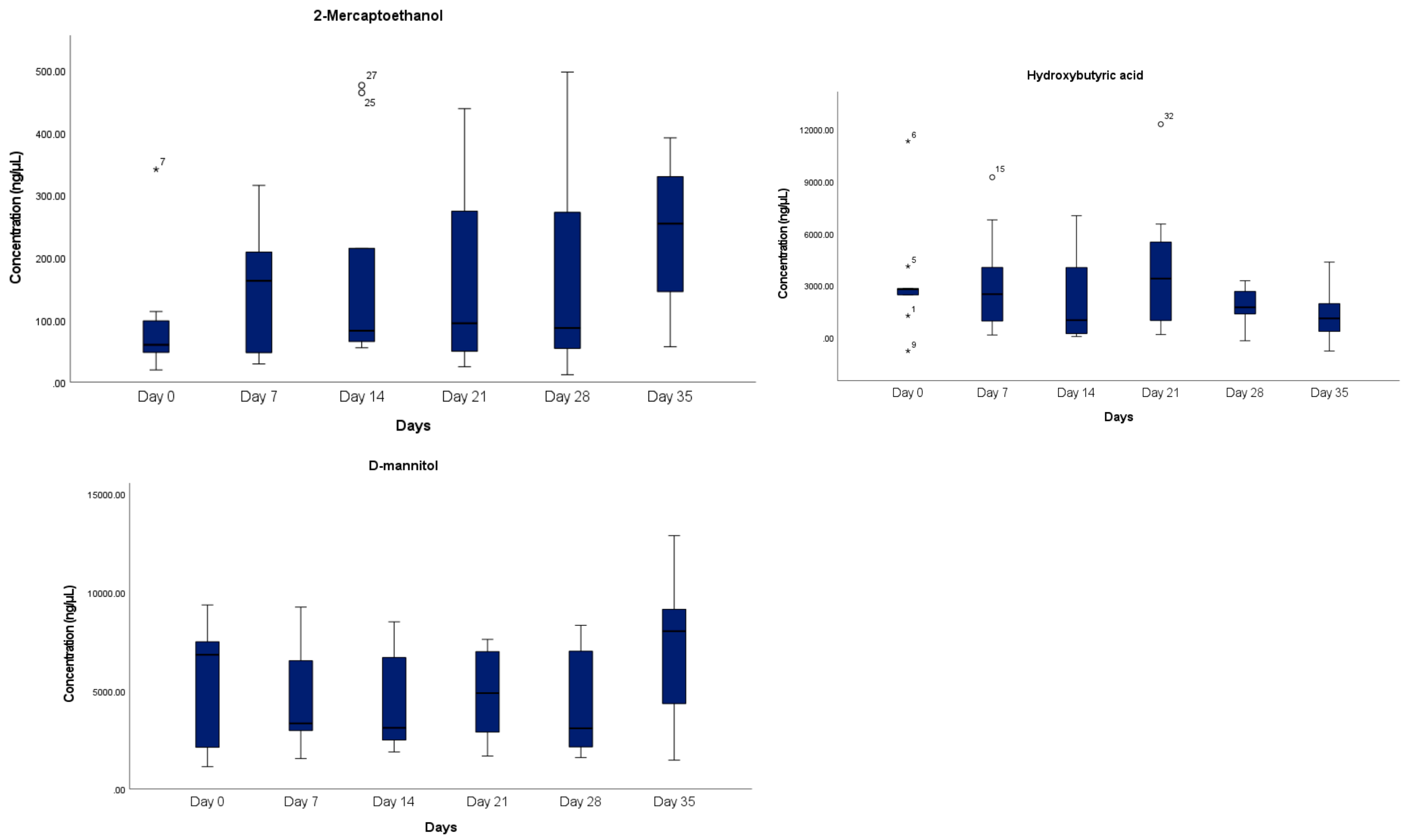

Supplementary Figure S1. Changes in the relative concentration of the identified potential biomarkers

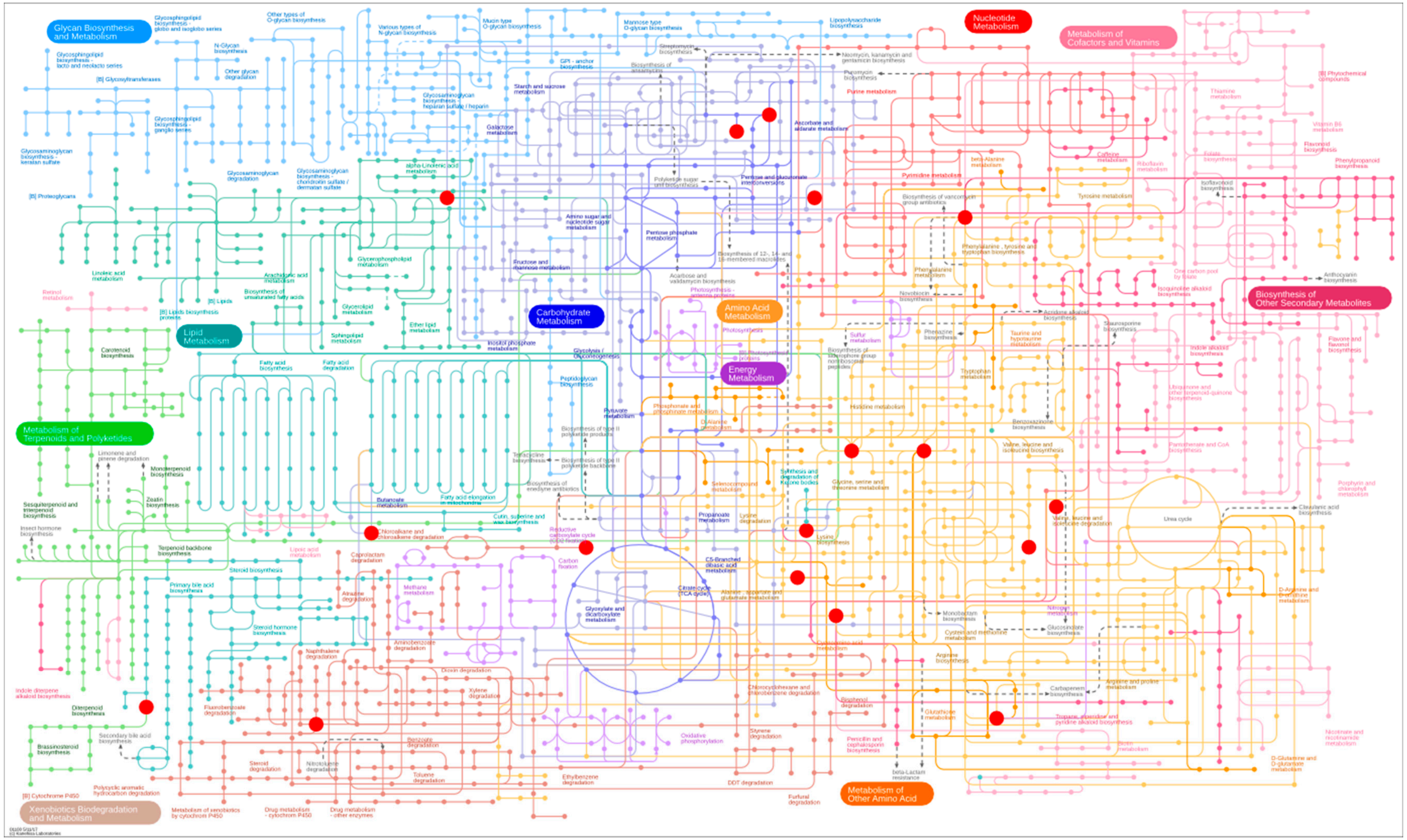

Supplementary Figure S2. KEGG pathways map; the highlighted lines indicate the metabolic pathways associated with the all the identified differentially expressed metabolites markers. *Colored circles indicate the metabolic biomarkers.

Table S1. Quantitative comparison of the additional potential biomarkers from the serum samples of TY2D induced SD-rats and control group.

Metabolite	VIP	FC	Regulation	P-value	Metabolite	VIP	FC	Regulation	P-value
Phenobarbital	2.6334	0.032928	Down	4.73E-05		L-Lysine	2.0466	Up	0.005064
1,2-Ethenediol	2.6259	0.38335	Down	5.02E-05		Butanediol	2.0327	Down	0.005069
L-Serine	2.6153	4.8654	Up	5.46E-05		Diethanolamine	2.0167	Down	0.005506
D-Lyxose	2.6035	0.34936	Down	5.98E-05		Ethanediol	2.0059	Down	0.007026
Heptasiloxane	2.596	0.21156	Down	6.34E-05		Glycine	1.9978	Up	0.007047
Butanediol	2.512	0.44307	Down	0.000183		Trimethylsiloxyvaleric acid	1.9953	Down	0.007416
L-Rhamnose	2.4514	0.39137	Down	0.000184		3-Amino-2-piperidone	1.989	Up	0.008092
Allocholic acid	2.4506	0.22565	Down	0.000266		Erythro-Pentonic acid	1.9792	Down	0.008412
Phenylmorphobutanoate	2.4132	2.4033	Down	0.000294		L-Norleucine	1.9579	Down	0.008829
Salicylic acid	2.3973	0.4332	Down	0.000295		Glycolic acid	1.9425	Up	0.012317
D-Fructose	2.3825	0.43966	Down	0.000542		á-D-Glucopyranose	1.9274	Down	0.014649
Campesterol	2.3821	0.20141	Down	0.000941		L-Valine	1.9218	Down	0.015356
Methyl galactoside	2.3585	0.11678	Down	0.001035		Glycerol	1.9086	Up	0.016429
Gluconic acid	2.3148	0.15894	Down	0.001075		L-Norvaline	1.9063	Down	0.016845
3-Hydroxy-3-phenylpropanoic acid	2.2994	0.31427	Down	0.001609		Pyrazine	1.5065	Up	0.017551

D-glucopyranoside	2.2889	0.44989	Down	0.001917	Phenoxy phenylamine	1.5062	Up	0.021457
L-tyrosine	2.1996	2.4318	Up	0.001982	Ribitol	1.4968	Down	0.025419
Hydroxy norvaline	2.1838	2.244	Up	0.002107	Silane	1.5062	Up	0.027944
L-Asparagine	2.1774	2.0191	Up	0.002113	Propanoic acid	1.4968	Down	0.027978
Octanoic acid	2.1744	2.0161	Up	0.002162	D-Pinitol	1.8978	Down	0.029596
Hydroxy isovaleric acid	2.1588	0.48178	Down	0.002265	Difluoro-3-methylbenzoic acid	1.8925	Up	0.032278
Hexenoic acid	2.1216	11.363	Up	0.002443	L-Leucine	1.8923	Down	0.032966
Galactaric acid	2.1079	2.1286	Up	0.002663	2-Pyridinemethanol	1.8848	Up	0.035659
D-Ribofuranose	2.0768	0.30727	Down	0.002822	Amino caprylic acid	1.878	Up	0.035929
L-Glutamine	2.0708	2.0854	Up	0.002947	Butanoic acid	1.8756	Down	0.037041
Butanoic acid	2.0597	0.37117	Down	0.002986	Pentitol	1.8672	Down	0.039723
Aminobutyric acid	2.0592	2.0231	Up	0.004248	Hydroxybutyric acid	1.8645	Down	0.046603
2-Butene-1,4-diol	2.0551	0.4307	Down	0.00467	Triethylene glycol	1.8596	Up	0.047395

Supplementary, Table S2. Metabolic pathways most affected during diabetes in serum samples from pathway-impact analysis with metaboAnalyst 5.0.

Pathway Name	Match Status	p	Impact
Aminoacyl-tRNA biosynthesis	8/48	**9.0531E-7**	**0.16667**
Valine, leucine, and isoleucine biosynthesis	2/8	0.0082418	0.0
Glyoxylate and dicarboxylate metabolism	3/32	**0.018449**	0.14815
Phenylalanine, tyrosine, and tryptophan biosynthesis	1/4	0.07039	0.5
Alanine, aspartate, and glutamate metabolism	2/28	**0.089135**	0.11378
D-Glutamine and D-glutamate metabolism	1/6	0.10377	0.0
Nitrogen metabolism	1/6	0.10377	0.0
Glycine, serine, and threonine metabolism	2/33	0.11788	0.46284
Ubiquinone and other terpenoid-quinone biosynthesis	1/9	0.15168	0.0
Valine, leucine, and isoleucine degradation	2/40	0.16139	0.0
Phenylalanine metabolism	1/10	0.16709	0.0
Biotin metabolism	1/10	0.16709	0.0
Arginine biosynthesis	1/14	0.22609	0.0
Butanoate metabolism	1/15	0.2402	0.0
Glycolipid metabolism	1/16	0.25406	0.23676
Pantothenate and CoA biosynthesis	1/19	0.29419	0.0
Sphingolipid metabolism	1/21	0.31978	0.0
Pentose phosphate pathway	1/22	0.33224	0.04712
Propanoate metabolism	1/23	0.34447	0.0
Lysine degradation	1/25	0.3683	0.0
Galactose metabolism	1/27	0.39129	0.0
Glutathione metabolism	1/28	0.40248	0.08873
Porphyrin and chlorophyll metabolism	1/30	0.42427	0.0
Cysteine and methionine metabolism	1/33	0.45553	0.02184
Amino sugar and nucleotide sugar metabolism	1/37	0.49466	0.0
Fatty acid degradation	1/39	0.5132	0.0
Pyrimidine metabolism	1/39	0.5132	0.0
Steroid biosynthesis	1/42	0.53978	0.0
Tyrosine metabolism	1/42	0.53978	0.13972
Primary bile acid biosynthesis	1/46	0.57305	0.00758
Purine metabolism	1/65	0.7019	0.0

Supplementary Table S3: Metabolic pathways and the metabolite involved from MetPA analysis**.**

Pathway	Metabolites involved
Ammonia Recycling	Glycine, L-Asparagine, L-Serine, L-Glutamine
Carnitine Synthesis	Glycine; L-Lysine
Amino Sugar Metabolism	L-Glutamine; D-Fructose
Aspartate Metabolism	L-Asparagine, L-Glutamine

Galactose Metabolism	Glycerol; D-Fructose
Propanoate Metabolism	Propionic acid; L-Valine
Methionine Metabolism	Glycine; L-Serine
Biotin Metabolism	L-Lysine
Phenylacetate Metabolism	L-Glutamine
Homocysteine Degradation	L-Serine
Glutamate Metabolism	Glycine
Phosphatidylethanolamine Biosynthesis	L-Glutamine
Thyroid hormone synthesis	L-Serine
Glycine and Serine Metabolism	Glycine; L-Serine
Valine, Leucine, and Isoleucine Degradation	L-Leucine; L-Valine
Vitamin K Metabolism	Propionic acid
Alanine Metabolism	Glycine
Butyrate Metabolism	Butyric acid
Catecholamine Biosynthesis	L-Tyrosine
Purine Metabolism	Glycine; L-Glutamine
Glutathione Metabolism	Glycine
Glycerolipid Metabolism	Glycerol
Phenylalanine and Tyrosine Metabolism	L-Tyrosine
Selenoamino Acid Metabolism	L-Serine
Urea Cycle	L-Glutamine
Lysine Degradation	L-Lysine
Starch and Sucrose Metabolism	D-Fructose
Fructose and Mannose Degradation	D-Fructose
Fatty Acid Biosynthesis	Butyric acid
Nicotinate and Nicotinamide Metabolism	L-Glutamine
Porphyrin Metabolism	Glycine
Sphingolipid Metabolism	L-Serine
Arginine and Proline Metabolism	Glycine
Warburg Effect	L-Glutamine
Pyrimidine Metabolism	L-Glutamine
Bile Acid Biosynthesis	Glycine
Tyrosine Metabolism	L-Tyrosine
